# Role of Perturbated Hemostasis in MASLD and Its Correlation with Adipokines

**DOI:** 10.3390/life14010093

**Published:** 2024-01-07

**Authors:** Salvatore Pezzino, Tonia Luca, Mariacarla Castorina, Stefano Puleo, Saverio Latteri, Sergio Castorina

**Affiliations:** 1 Mediterranean Foundation “GB Morgagni”, 95125 Catania, Italymariacarlacastorina@gmail.com (M.C.); sergio.castorina@unict.it (S.C.); 2 Department of Medical, Surgical Sciences and Advanced Technologies “G.F. Ingrassia”, University of Catania, 95123 Catania, Italy; saverio.latteri@unict.it

**Keywords:** dysfunctional hemostasis, NAFLD, MASLD, adipokines, coagulation, platelet, endothelium, inflammation, insulin resistance

## Abstract

The prevalence of metabolic dysfunction-associated steatotic liver disease (MASLD) continues to rise, making it one of the most prevalent chronic liver disorders. MASLD encompasses a range of liver pathologies, from simple steatosis to metabolic dysfunction-associated steatohepatitis (MASH) with inflammation, hepatocyte damage, and fibrosis. Interestingly, the liver exhibits close intercommunication with fatty tissue. In fact, adipose tissue could contribute to the etiology and advancement of MASLD, acting as an endocrine organ that releases several hormones and cytokines, with the adipokines assuming a pivotal role. The levels of adipokines in the blood are altered in people with MASLD, and recent research has shed light on the crucial role played by adipokines in regulating energy expenditure, inflammation, and fibrosis in MASLD. However, MASLD disease is a multifaceted condition that affects various aspects of health beyond liver function, including its impact on hemostasis. The alterations in coagulation mechanisms and endothelial and platelet functions may play a role in the increased vulnerability and severity of MASLD. Therefore, more attention is being given to imbalanced adipokines as causative agents in causing disturbances in hemostasis in MASLD. Metabolic inflammation and hepatic injury are fundamental components of MASLD, and the interrelation between these biological components and the hemostasis pathway is delineated by reciprocal influences, as well as the induction of alterations. Adipokines have the potential to serve as the shared elements within this complex interrelationship. The objective of this review is to thoroughly examine the existing scientific knowledge on the impairment of hemostasis in MASLD and its connection with adipokines, with the aim of enhancing our comprehension of the disease.

## 1. Introduction

Recently, the classification of non-alcoholic fatty liver disease (NAFLD) and non-alcoholic steatohepatitis (NASH) has been revised to provide more precise and accurate descriptions of these disorders [1]. The revised categorization system suggests the utilization of metabolic dysfunction-associated steatotic liver disease (MASLD) and metabolic dysfunction-associated steatohepatitis (MASH) as appropriate terms [1].

The prevalence of MASLD continues to rise, making it one of the most prevalent chronic liver disorders [2,3]. The global incidence of MASLD is estimated to be 47 cases per 1000 individuals in the population, with a higher prevalence observed among males compared to females [2,3]. MASLD encompasses a range of liver pathologies, from simple steatosis to MASH with inflammation, hepatocyte damage, and fibrosis [4,5,6]. These pathological conditions are frequently correlated with several physiological alterations [7,8] and with obesity or overweight [5,9], both of which are established risk factors for the development of MASLD [10]. In fact, epidemiological studies have demonstrated that the prevalence of MASLD in individuals who are overweight ranges from 22.5% to 44.0% [10]. Conversely, the prevalence of MASLD among obese individuals can reach as high as 90% [10,11,12,13]. However, MASLD disease is a multifaceted condition that affects various aspects of health beyond liver function, including its impact on hemostasis [14,15,16]. Several studies suggest that alterations in coagulation parameters, the fibrinolytic process, and endothelial and platelet functions may play a role in the increased vulnerability and severity of MASLD [14,15,16,17,18,19]. This is not unexpected since the liver fulfills a crucial function in the production of coagulation factors and the maintenance of hemostatic equilibrium [17,20,21]. On the other hand, the liver exhibits close intercommunication with adipose tissue [22,23]. Interestingly, adipose tissue may contribute to the etiology and progression of MASLD by delivering free fatty acids derived from triglyceride lipolysis through the bloodstream to the liver [24] and also acting as an endocrine organ that releases several hormones and cytokines [25,26,27], with adipokines assuming a pivotal role [23,28,29]. Adipokines are involved in regulating energy expenditure, inflammation, and fibrosis in obesity and MASLD [30,31]. Numerous clinical trials have demonstrated altered serum profiles of adipokines in patients with MASLD [30,32]. There is increasing interest in the role of imbalanced adipokines as significant contributors to the altered hemostatic mechanism in MASLD. Gradually, adipokines are being recognized as significant contributors to the dysregulated hemostatic mechanism in MASLD [30,32]. For example, Dalbeni et al. (2022) recently found a positive correlation between the severity of MASLD/MASH and the levels of the hormone leptin, as well as an increase in platelet activation and aggregation, thereby potentially leading to the facilitation of arterial thrombosis [33].

MASLD is associated with increased levels of inflammation [32,34], which are frequently associated with insulin resistance (IR) [35,36,37,38,39]. IR could result in altered serum levels and the activity of adipokines [35,40], which could directly impact hemostasis [41,42,43,44] or indirectly affect it through the induction of augmented inflammation and IR [33,36,45,46]. The interplay between these factors results in a vicious cycle that perpetuates the development and progression of MASLD [33,36,45,46]. One of the effects of MASLD-exacerbated inflammation related to hemostasis may be the alteration of platelet function. Platelets’ uncontrolled aggregation contributes to thrombotic risk [33,45] and causes damage to the vascular endothelium [14,47,48]. The endothelium, a monolayer of specialized cells lining the interior surface of blood vessels, plays a crucial role in maintaining vascular health [47]. A healthy endothelium possesses antithrombotic properties that hinder the formation of blood clots [47]. Conversely, when endothelial function is compromised, it may lose its protective properties, thereby promoting thrombosis [14,47,48,49].

**Figure 1:**
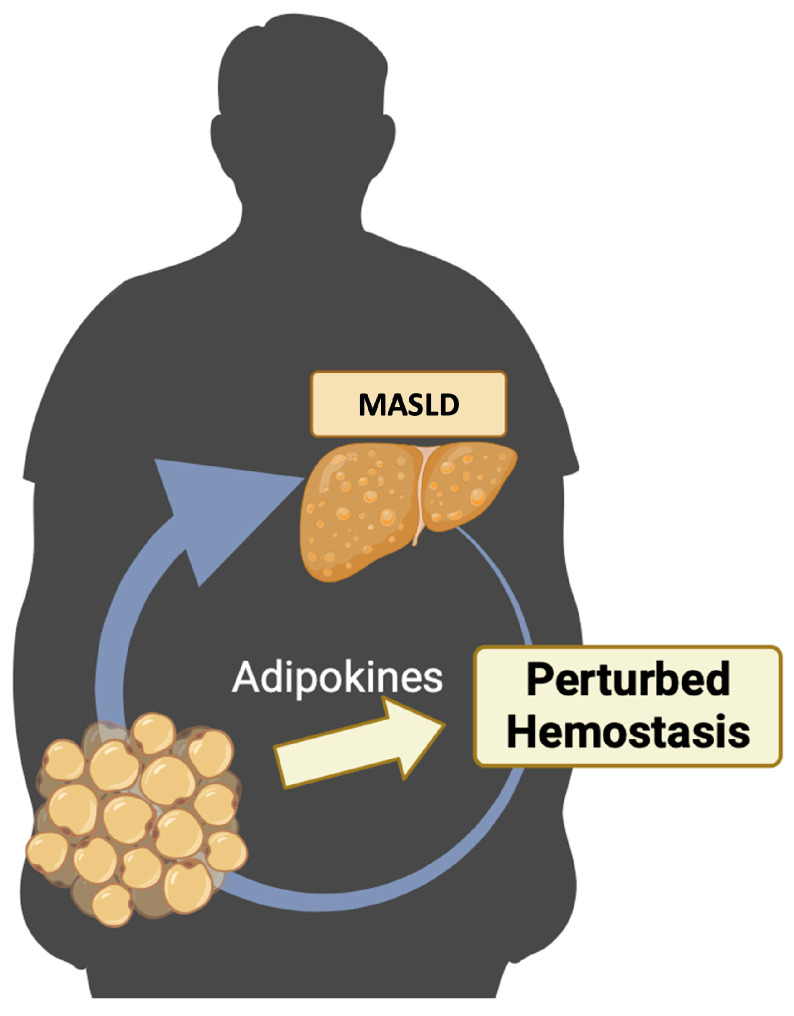
MASLD, hemostasis, and adipokines. Patients with MASLD have modifications in the physiological process of hemostasis. Adipokines, principally produced by the adipose tissue, could play a significant role in the pathogenesis of MASLD and could contribute to the disruption of hemostasis. Created with https://BioRender.com (accessed on 27 November 2023); MASLD: metabolic dysfunction-associated steatotic liver disease.

The purpose of this review is to conduct an exhaustive examination of the existing scientific literature concerning the disruption of hemostasis in MASLD and its association with adipokines (Figure 1).

## 2. Abnormal Hemostasis in Metabolic Dysfunction-Associated Steatotic Liver Disease (MASLD)

**Figure 2:**
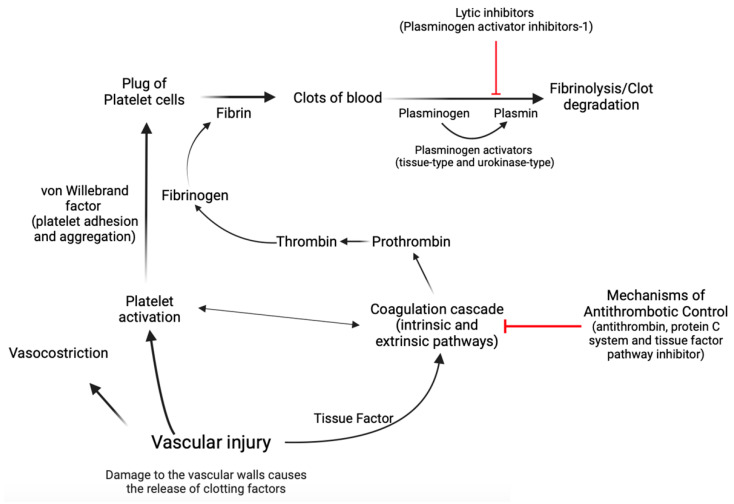
A simplified diagram illustrating the process of hemostasis. Hemostasis is a complex physiological process that relies heavily on the intricate interplay between coagulation factors that facilitate clot formation and those that promote clot dissolution. The maintenance of this delicate equilibrium is of paramount importance for the prevention of excessive bleeding or thrombosis and thus forms a critical aspect of the clinical management of many medical conditions. Created with https://BioRender.com (accessed on 29 November 2023).

Hemostasis is a complex biological process marked by a series of interconnected, sequential events that finally culminate in the formation of a “thrombus,” which obstructs the wounded area of the vascular structure, effectively controlling the bleeding [15,50,51,52,53]. Concisely, the first stage, known as primary hemostasis, encompasses not only vascular constriction but also the aggregation of platelets at an injury site to stop initial blood loss [50]. In the second stage of hemostasis, known as secondary hemostasis, the coagulation cascade is activated, which involves a series of reactions that result in the conversion of soluble fibrinogen into insoluble fibrin, forming a stable clot that reinforces the platelet plug [15,50,51,52,53]. During the coagulation cascade, several protein factors and enzymes are sequentially activated to form a clot. This cascade involves both the intrinsic and extrinsic pathways, which eventually merge to activate the final common pathway [15,50,51,52,53]. Factors within the blood itself start the intrinsic pathway of the coagulation cascade. These factors include factors XII, XI, IX, and VIII, whose activation leads to the production of thrombin, which plays a central role in clot formation [15,50,51,52,53]. The extrinsic pathway of the coagulation cascade is initiated by tissue factor, which is released from damaged tissues and triggers a series of reactions that activate factor VII and ultimately lead to the production of thrombin [15,50,51,52,53]. The activation of thrombin in both the intrinsic and extrinsic pathways leads to the conversion of fibrinogen to fibrin [15,50,51,52,53]. Endothelial cells exert control over blood fluidity and tissue perfusion due to their strategic location at the interface between blood and tissues [15,50,51,52,53]. Additionally, these cells play a crucial role in guiding inflammatory cells to specific regions that require defense or repair [15,50,51,52,53]. Endothelial cells mitigate coagulation by the release of tissue factor and thrombin inhibitors, as well as receptors that facilitate protein C activation [15,50,51,52,53]. Protein C acts as a natural anticoagulant [15,50,51,52,53], preventing excessive clot formation by limiting thrombin production. Plasminogen activator inhibitor-1 (PAI-1) has a crucial role in coagulation and fibrinolysis processes [54,55]. It is an inhibitor of tissue plasminogen activator and urokinase-type plasminogen activator, which are enzymes responsible for the conversion of plasminogen into plasmin [54,55,56]. Plasmin is an enzyme that helps to dissolve fibrin in blood clots, thereby aiding in clot resolution [54,55,56]. In the context of coagulation, an increase in PAI-1 could lead to a decrease in fibrinolysis as there would be less active plasmin to break down fibrin in the clots [54,55,56]. In contrast, von Willebrand factor (vWF) is an essential glycoprotein that facilitates platelet adhesion and aggregation [15,50,51,52,53]. Acting as a bridge between platelets and damaged blood vessel walls, vWF promotes the formation of stable clots during hemostasis. In cases of endothelial dysfunction or cardiovascular diseases, the balance between protein C and vWF can be disrupted, leading to an increased risk of thrombosis [57] (Figure 2).

Metabolic dysfunction-associated steatotic liver disease (MASLD) is a complex disorder that has implications for several aspects of health beyond the functioning of the liver. According to previous research, this includes its impact on hemostasis [15,17,51,58,59,60,61]. In fact, multiple studies have indicated that changes in coagulation parameters, the fibrinolytic process, and the activities of endothelial cells and platelets may contribute to the heightened susceptibility and severity of MASLD [12,13,14,15,18,19].

The significance of coagulation, platelet, and endothelial dysfunctions in the pathogenesis of MASLD will be discussed in the following sections.

### 2.1. Coagulation Dysfunctions

MASLD dysfunctional coagulation has been the focus of several studies [16,18,62]. The coexistence of coagulation abnormalities in individuals with MASLD/metabolic dysfunction-associated steatohepatitis (MASH) highlights the intricate relationship between liver dysfunction and the mechanism of hemostasis [17,58]. Within the framework of metabolic dysfunction, it was reported that hepatic damage could contribute to the initiation of coagulation, hence inducing the initiation of fibrogenesis [28,58,63] and vice versa [16,18,62,64]. The liver plays a significant role in the regulation of hemostatic balance and in the synthesis of coagulation factors, which have been shown to diminish as liver fibrosis advances [65]. Moreover, hepatocytes have a role in the post-translational modification of coagulation factors [65]. Therefore, the elevated occurrence of thrombotic events and changes in coagulation time observed in individuals with MASLD can be attributed to the compromised hepatic production of coagulation factors [17,20,60].

The presence of a hypercoagulable state in MASLD individuals is supported by blood-elevated levels of prothrombotic factors, including prothrombin and fibrinogen. These prothrombotic factors play a role in the initiation and advancement of blood clot formation [15,50,51,52,53]. This suggests that an aberrant coagulation cascade may be at play in MASLD individuals, predisposing them to thrombotic complications [16,18,62]. In addition, the presence of elevated levels of D-dimer, a marker that indicates the formation and breakdown of fibrin, provides further support for this association [58]. Given the growing incidence of MASLD in the general population, these findings underscore the importance of vigilant thromboprophylaxis in affected individuals [16,58]. Individuals suffering from MASLD exhibit heightened levels of factor VIII and reduced levels of protein C [62]. Valenti et al. revealed an independent association between liver fibrosis indices and an elevated Factor VIII/protein C ratio in MASLD patients, suggesting the presence of the procoagulant state [63]. Additionally, they found genetic mutations, mainly the PNPLA3 p.I148M variant, that were linked to the levels of change in factor VIII and protein C [63]. This discovery further supports the idea that liver damage significantly alters the balance of coagulation [63]. In MASLD patients, there is also an alteration of the levels of antithrombin and von Willebrand factor (vWF) [66,67], whose altered activation of the latter leads to the dysregulated formation of a platelet plug [66]. The severity of alteration in hemostasis was more pronounced in patients with advanced liver disease such as MASH or cirrhosis [68,69], in which more advanced hepatocellular damage is present [59]. Hepatocellular damage of a significant degree could result in a compromised production of various coagulation factors, including fibrinogen; thrombin; and factors V, VII, IX, and X. These factors are predominantly synthesized in the liver [68,69]. As a result, this gives rise to an increased vulnerability to both thrombotic and hemorrhagic events [68,69].

The occurrence of insulin resistance (IR) is notably elevated in individuals with MASLD and further exacerbated in patients diagnosed with MASH [21,37,70,71]. Liver triglyceride accumulation, increased lipolysis, and increased synthesis of triglycerides are all linked to IR. This accumulation of fat in the liver can have detrimental effects on clotting dynamics [21,37,70,71]. Moreover, the presence of visceral fat tissues in MASLD patients is directly correlated with IR and impaired clotting dynamics [72]. The activation of the coagulation cascade may occur as a result of IR, which could be characterized by elevated blood levels of plasminogen activator inhibitor-1 (PAI-1) [61,72,73]. It is known that PAI-1 has a crucial role in coagulation and fibrinolysis processes [54,55]. The erratic pattern of PAI-1 blood levels was correlated with an imbalance of the hemostatic system, leading to the occurrence of bleeding or thrombotic problems [54,55] and cardiovascular disease [74]. Excessive levels of PAI-1 were observed in individuals with metabolic syndromes, such as MASLD [75,76]. Furthermore, cross-sectional studies have provided data indicating connections between elevated plasma levels of PAI-1 and the presence and severity of MASLD in human subjects [57,77]. Evidence has highlighted that PAI-1 increases the likelihood of thrombosis and could accelerate the progression of liver disease as a result of local tissue ischemia caused by intrahepatic thrombi [78,79]. On the other hand, PAI-1 is an essential regulator of lipid metabolism in the liver [56], and enhanced levels of PAI-1 in the serum are correlated with adult MASLD pathogenesis beyond its function in hemostasis [75,77]. These scientific findings demonstrate the convergence of the metabolic pathway and hemostasis in MASLD patients.

Alteration in coagulative mechanisms contributes to an increased occurrence of atherosclerosis and thrombotic events, both of which are prevalent in MASLD patients and are associated with a heightened risk of cardiovascular disease and mortality [80,81]. The recent results of a meta-analysis have corroborated the positive and statistically significant correlation that exists between MASLD and portal vein thrombosis [82]. The presence of a hypercoagulable state in patients with MASLD has the potential to result in the formation of microthrombi in the hepatic veins and arteries, leading to disrupted blood flow [58]. Increased rates of clinically significant thrombotic events, such as pulmonary embolism, deep vein thrombosis, and portal vein thrombosis, are explicable by abnormalities at all stages of hemostasis [83,84], including a condition of hypercoagulation derived from decreased levels of PAI-1 [61,72,73].

### 2.2. Dysfunction of Platelets

Altered blood platelet activity was involved in the pathogenesis of MASLD [33]. Shin et al. have examined the association between platelet count and MASLD within a sizable population-based cohort. They discovered a negative correlation between platelet count and the occurrence of MASLD [85]. This correlation remained significant even after controlling for potential confounding variables, including age, gender, body mass index, and metabolic parameters [85]. The study conducted by Madan et al. (2016) revealed that individuals with MASLD have enhanced mean platelet volume (MPV), an index that serves as an indicator of both platelet size and activation [86]. This finding suggests heightened platelet activity in MASLD patients [86]. Saremi et al. (2017) reported a substantial correlation between platelet count and MPV with the severity of fibrosis in patients with MASLD [87] and its progression in a more severe form [88]. Furthermore, the number of platelets observed in the liver sinusoids is correlated with the incidence of ballooning degeneration (a form of damage to hepatocytes) in the liver [89] and the extent of fat infiltration [88]. For this reason, the platelet count is included in scoring systems utilized in clinical settings for the purpose of prognosticating the advancement and gravity of MASH [90] and as a predictive factor for the occurrence of hepatocellular carcinoma in individuals diagnosed with MASLD [91].

The association between MASLD and impaired platelet function can be attributed to multiple contributing factors. As mentioned, elevations in fibrinogen, together with a reduction in antithrombin, were reported in individuals with MASLD [92]. Thrombin is widely recognized as a potent activator of platelets, functioning by cleaving the protease-activated receptors that are expressed on human platelets [93]. Moreover, fibrinogen plays a significant role in facilitating platelet aggregation [94]. The platelet hyperactivation, combined with the prothrombotic factors mentioned earlier, increases the risk of clot formation and thrombotic events in MASLD patients. In addition, the advancement of liver fibrosis results in an enlargement of the spleen and a decrease in thrombopoietin levels, subsequently causing a reduction in platelet count [95,96].

The pathogenesis of MASLD may encompass the involvement of lysosomal acid lipase (LAL), an enzyme that breaks down triglycerides and cholesteryl esters in various hepatic cells and monocyte-macrophages produced from bone marrow [97]. A reduction in platelet LAL levels has been linked to the severity of MASLD in humans [98]. The process of lipophagy was enhanced, leading to an accumulation of cholesterol. This, in turn, stimulated the activation of platelet metabolism, migration, and aggregation, ultimately resulting in the enhancement of their pro-inflammatory capacity [99,100]. Furthermore, the occurrence of systemic inflammation has the potential to induce platelet activation and aggregation [33,101]. This assertion is substantiated by research indicating elevated concentrations of proinflammatory cytokines, including interleukin-6 and tumor necrosis factor-alpha, in individuals diagnosed with MASLD [102,103]. These cytokines have the potential to directly impact the functioning of platelets [102,103]. Furthermore, bone marrow adiposity, frequently observed in subjects with metabolic syndrome and obesity, directly affects platelets [104]. Megakaryocyte maturation was increased in medullar adiposity, leading to increased thrombogenicity and activation of platelets, thus potentially providing a feed-forward loop of hepatic platelet aggregation in MASH [104].

### 2.3. Endothelial Dysfunction

Endothelial dysfunction (ED) has been extensively documented in MASLD, which was correlated with a heightened incidence of augmented carotid wall intimal thickness, atherosclerotic plaques, and elevated concentrations of indicators relating to ED [105,106]. Multiple investigations have provided evidence of the existence of impaired sinusoidal endothelial function within the hepatic microcirculation, observed in both the initial phases of MASLD and more advanced stages like cirrhosis [14,48,49,107]. The activation of hepatic stellate cells and Kupffer cells depends on the dysfunction of hepatic sinusoidal endothelial cells [108,109]. The production of several prothrombotic substances and receptors, along with the recruitment of neutrophils and platelets, promotes the creation of sinusoidal microthrombus [110]. This process leads to the destruction of parenchymal tissue and the advancement of fibrosis [110]. The extent of ED is directly related to the severity of MASLD and serves as an indicator of the severity of damage to the vascular walls [111,112]. Therefore the ED may contribute to the deterioration of coagulation disorders by promoting platelet activation and aggregation, causing additional harm to the endothelium [113,114]. In their recent study, Ogresta et al. (2022) found an interesting link between ED in the systemic and portal venous circulation of people with MASLD and platelet activation and aggregation [14]. The endothelium exhibits significant involvement in the metabolic utilization of long-chain fatty acids as energy sources while also possessing a multitude of regulatory capabilities [47,115]. This assertion is substantiated by scientific investigations, which indicate that the presence of ED is correlated with a heightened susceptibility to cardiovascular illnesses and preliminary indications of atherosclerosis among individuals diagnosed with MASLD [116,117]. Accordingly, it was suggested that incorporating exercise training of moderate intensity into the therapeutic regimen may aid in ameliorating the ED and mitigating the propensity for cardiovascular disease that is emblematic of MASLD [116,117]. The impairment of endothelium in MASLD is not confined solely to the blood vessels within the liver but rather encompasses additional vascular networks [14,48,118]. Research findings have indicated that MASLD exhibits a correlation with an elevated susceptibility to cerebrovascular complications [9,119]. This association subsequently contributes to the occurrence of neurodegenerative alterations within the brain and an augmented likelihood of developing dementia [120,121]. Critical to physiological nitric oxide (NO) production is endothelial nitric oxide synthase (eNOS), an enzyme that is primarily found in the endothelium of blood vessels [122]. Impaired eNOS activity occurs in insulin resistance (IR), leading to decreased generation of NO and, consequently, ED [123]. Research has demonstrated a reciprocal relationship between ED and IR, linking both to cardiovascular and metabolic disorders [123,124]. Furthermore, insulin and inflammation impact vascular homeostasis by stimulating the production of NO, which helps preserve the health of the endothelium through its anti-inflammatory and antithrombotic properties [123,124].

The precise mechanisms elucidating ED in MASLD remain incompletely comprehended. Nonetheless, it has been associated with IR, lipid dysmetabolism, chronic inflammation, and elevated levels of fatty acids, which collectively contribute to the impairment of ED observed in individuals diagnosed with MASLD [48,113,114,118].

### 2.4. Hemostasis Dysfunction in MASLD Patients during COVID-19: Recent Learning from the Pandemic

In order to substantiate the thesis that changes in hemostatic mechanisms play a crucial role in the etiopathogenesis of MASLD and its progression into more severe manifestations, we can refer to the data acquired during the recent COVID-19 pandemic.

Numerous investigations have elucidated the correlation existing between MASLD and the degree of severity observed in cases of COVID-19. Portincasa et al. (2020) revealed the convergence of COVID-19 and MASLD as two inter-related pandemics [125,126]. Singh et al. (2021) executed an all-inclusive systematic review and meta-analysis, which discovered a noteworthy correlation between MASLD and clinical outcomes in individuals afflicted with COVID-19 [127]. On the other hand, there is a probable connection between diabetes mellitus and obesity and the pathophysiology of COVID-19, as well as with particular abnormalities in liver pathology [128]. Recent findings by Miranda et al. (2023) provide updated insights into the correlation between liver injury and MASLD in subjects affected by COVID-19 [129]. Other investigations have revealed that MASLD serves as a prognostic indicator for hepatic impairment in individuals admitted to healthcare facilities due to COVID-19 infection [130,131]. Interestingly, using a systems biology approach has disclosed a shared molecular basis for both COVID-19 and MASLD. This was achieved by successfully extracting 10 hub genes that could be used as new therapeutic targets for both diseases [132]. As for MASLD, changes in platelet count and functions, hypercoagulability, and hypofibrinolysis are all physiological and pathological features seen in people with severe SARS-CoV-2 infections [133,134,135]. These changes are closely associated with the initiation and progression of an immune-thrombo-inflammatory clinical presentation [133,134,135]. These characteristics exhibit clinical significance, leading to the occurrence of thrombosis in various anatomical regions [133,134,135,136]. A recent study by Abenavoli et al. (2023) discovered that individuals with a severe manifestation of COVID-19 exhibit a correlation between liver illness and changes in coagulative and fibrinolytic pathways [137]. Specifically, the researchers observed decreased levels of fibrinogen and increased levels of D-dimer, together with histological liver abnormalities. The available evidence indicates that fibrinogen and D-dimers have the potential to serve as prognostic indicators for assessing the degree of liver disease in individuals with COVID-19. This highlights the significant involvement of coagulation balance in patients experiencing severe manifestations of COVID-19 [137]. Moreover, it is usual to observe altered levels of PAI-1 during infection, which are often linked to a condition of reduced fibrinolysis and the occurrence of thrombotic problems. PAI-1 levels are basally elevated in patients with MASLD [57,77]; this may also account for the more severe hemostasis alterations observed in COVID-19 patients, which would increase the risk of mortality [129,131].

**Figure 3:**
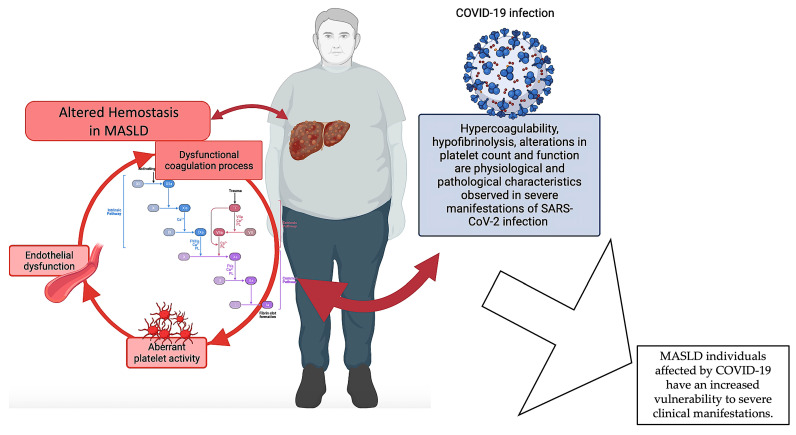
Dysfunctional hemostasis in MASLD. The pathophysiology of MASLD encompasses a complex interplay of various mechanisms, including coagulation abnormalities and platelet and endothelial dysfunction, which collectively contribute to the dysregulation of hemostasis in MASLD individuals. Severe manifestations of MASLD are more likely to occur in individuals with COVID-19, in which hemostasis disruptions appear to play a significant role. Given that liver malfunctioning is a common disturbance between the two diseases, it is possible that the coexistence of COVID-19 disease, which is also distinguished by hemostasis alterations, could exacerbate the hemostatic dysfunction of individuals with MASLD. Created with https://BioRender.com (accessed on 27 November 2023) and modified with Microsoft PowerPoint v.16; MASLD: metabolic dysfunction-associated steatotic liver disease.

In summary, the available data indicate a correlation between MASLD and COVID-19, whereby MASLD is linked to a heightened susceptibility to severe manifestations in individuals affected by COVID-19 and where hemostasis alterations seem to play an important role. Given that liver malfunctioning is a common disturbance between the two diseases, it is possible that the coexistence of COVID-19 disease, which is also characterized by disruptions in hemostasis, could worsen the hemostatic dysfunction of individuals with MASLD. Nevertheless, additional investigation is warranted to comprehensively understand the fundamental biological processes and the ramifications of MASLD on COVID-19 outcomes (Figure 3).

## 3. Adipokines in Metabolic Dysfunction-Associated Steatotic Liver Disease (MASLD) and Their Role in Perturbated Hemostasis

Adipokines comprise a class of polypeptides that are predominantly produced and secreted by adipose tissue [32,138]. They play a crucial role in the regulation of hepatic insulin sensitivity [32,138,139]. Furthermore, adipokines have been implicated in the pathogenesis of metabolic syndrome and metabolic dysfunction-associated steatotic liver disease (MASLD) [32,34,45,138,139]. There is a growing interest in adipokines as regulators of the hemostasis process. Adipokines are increasingly being recognized as significant contributors to the dysregulated hemostatic mechanism in MASLD [30,32,41,42,43]. As previously stated, inflammation is frequently associated with MASLD and insulin resistance (IR) [32,34,35,36,37,38,39]. The IR was correlated to the elevation of blood glucose levels [140], which in turn might lead to the accumulation of fatty acids and triglycerides in the liver, causing the development of hepatic steatosis in MASLD [141,142]. The IR in MASLD was also correlated with changes in the activity and concentration of adipokines [35,40], which could directly alter hemostasis [41,42,43,44] or indirectly alter it by inducing, in a vicious circle, IR, hepatic steatosis, and hyperinflammation [33,36,45,46]. As a result, a surge in prolonged inflammation could lead to altered mechanisms of hemostasis [15,50,51,52,53] at different intercorrelated levels, which can be recapitulated as follows: (1) increased platelet activation resulting from heightened von Willebrand factor levels, consequently elevating the risk of thrombosis [24,36]; (2) hypercoagulability due to augmented Factor VIII and fibrinogen levels; decreased levels of the anticoagulants antithrombin and protein C, dropping the hemostatic balance toward clotting [15,50,51,52,53]; (3) increased levels of PAI-1 while tissue activating factor antigen and tissue plasminogen activator decrease, resulting in a chronic state of hypofibrinolysis [15,50,51,52,53]; and (4) endothelial dysfunction, also thereby increasing the risk of thrombosis [142,143].

**Figure 4:**
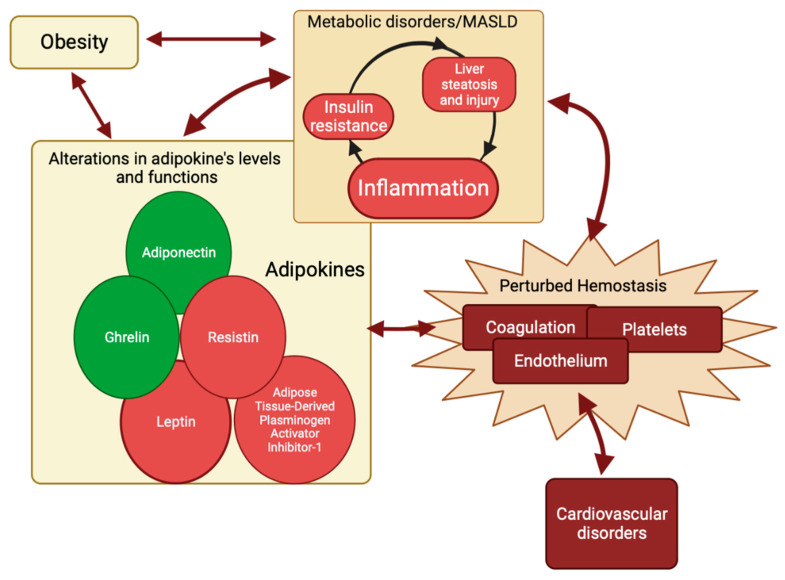
Circuit of perturbed hemostasis. Metabolic inflammation and liver injury are integral components of MASLD. The intricate interplay between these components and the hemostasis is distinguished by mutual influences and the elicitation of alterations. Adipokines have the potential to function as the common factors within this intricate association. Adiponectin and ghrelin (circles in green) have a favorable impact on MASLD’s pathogenesis and hemostasis, while adipose tissue-derived plasminogen inhibitor-1, leptin, and resistin (circles in red) have a detrimental effect on MASLD. Created with https://BioRender.com (accessed on 27 November 2023); MASLD: metabolic dysfunction-associated steatotic liver disease.

To summarize, metabolic inflammation and liver injury are essential components of MASLD [144,145]. The interdependence of these components and the hemostasis pathway is characterized by reciprocal influences and the induction of alterations. Adipokines may serve as the shared elements within this complex relationship (Figure 4).

In the subsequent subsections, we will examine several adipokines implicated in the pathogenesis of MASLD and their demonstrated involvement in hemostasis mechanisms, as summarized in Table 1.

### 3.1. Adipose Tissue-Derived Plasminogen Activator Inhibitor-1

Platelets possess a significant reservoir of circulating plasminogen activator inhibitor-1 (PAI-1) [146]. Upon activation in response to vascular injury, platelets release this reservoir, effectively preserving the growing thrombus from premature dissolution by fibrinolysis [146,147]. Nevertheless, PAI-1 is also produced by various other cell types, including adipocytes [148]. In fact, adipose tissue, particularly from the abdominal region, can directly secrete the adipose tissue-derived plasminogen activator inhibitor-1 (ATDPAI-1) [149,150]. There is an increased emphasis on the detrimental impact of ATDPAI-1 on both physiological metabolism and vascular biology [148,149,150,151]. This effect is particularly pronounced in visceral fat, where ATDPAI-1 expression is largely observed [149,152]. The discovery of the association between the hemostatic and inflammatory pathways has revealed a specific function for ATDPAI-1 [103,153,154,155,156]. In fact, during the process of inflammation, a significant release of proinflammatory cytokines occurs [153,154,155,157]. These cytokines have a direct impact on the synthesis of ATDPAI-1 and result in an elevation of its levels in the bloodstream [158,159,160]. PAI-1, secreted by adipose tissue into the portal circulation, directly interacts with liver parenchymal and immune cells, leading to enhanced activation of pro-inflammatory cytokines and increased dysregulation of coagulation and fibrinolysis [44,159]. These interactions ultimately contribute to the permanent activation of pro-inflammatory pathways and the disruption of normal hemostatic balance [44,159]. The adipose tissue, especially in the inflamed state seen in MASLD, releases pro-inflammatory cytokines such as interleukin-6 [161] and tumor necrosis factor-α [157,162]. These cytokines, in turn, could induce the production of PAI-1 in human adipocytes in an autocrine way [160,163]. On the other hand, insulin resistance, which is associated with hyperinsulinemia in MASLD [38], could lead to the increase in PAI-1 plasma levels and PAI-1 gene expression in adipose tissue [164,165], therefore establishing a connection between both metabolic and inflammation pathways.

Therefore, ATDPAI-1 and coagulation seem linked in a complex regulatory cycle with potential implications for MASLD [149]. In fact, the regulation of ATDPAI-1 blood levels could be crucial for maintaining a delicate balance in the hemostatic process, allowing adequate clot formation to prevent hemorrhage but preventing excessive clot stability, which could lead to damaging thrombosis and cardiovascular diseases [149,166,167,168].

### 3.2. Adiponectin

Adiponectin, predominantly synthesized by adipocytes, plays a crucial role in various metabolic processes [169]. Several studies have reported an inverse relationship between plasma adiponectin levels and the accumulation of body fat, insulin resistance (IR), and diabetes [170,171]. Adiponectin exerts advantageous impacts on hepatic lipid metabolism and insulin sensitivity, hence potentially influencing the hemostatic equilibrium through the enhancement of metabolic well-being in individuals with MASLD [170,171,172]. Low levels of adiponectin have been consistently observed in individuals with MASLD [170,173,174]. This suggests that adiponectin may play a role in the pathogenesis of this disease [170,171,172]. It was also reported that the simultaneous presence of heightened concentrations of leptin and resistin (two adipokines that will be discussed in the next paragraphs), together with diminished levels of adiponectin, may potentially contribute to the advancement of MASLD [175,176]. This finding suggests that adiponectin could potentially contribute to the prevention of liver fibrosis, a condition frequently linked to coagulation abnormalities in individuals with MASLD.

The dysfunction of hepatic mitochondria is a defining characteristic of the progression of MASLD, although the underlying mechanisms remain unknown [177]. Considering the significant contribution of endothelial nitric oxide synthase (eNOS) to mitochondrial dynamics in various tissues, it has become a plausible candidate for mediating the maintenance of mitochondrial function in the liver of MASLD [178]. In this context, it was reported that adiponectin could play a protective role in the pathogenesis of MASLD, reducing the inflammation by the inhibition of hepatic stellate cells and by upregulating the expression of eNOS [172,179,180,181].

Adiponectin has been shown to have a key role in hemostasis. Kato et al. (2006) found that adiponectin could function as an endogenous antithrombotic factor [182]. In adiponectin knockout mice, adiponectin deficiency increased thrombus development and platelet aggregation, which were reduced after adiponectin supplementation via adenovirus [182].

It was reported that altered levels of adiponectin could lead to altered levels of plasminogen activator inhibitor-1 (PAI-1) [42,183]. As aforementioned, PAI-1 is the primary blocker of plasminogen activator in plasma; increased levels of PAI-1 could lead to excessive blocking of tissue-plasminogen activator, leading to decreased clot breakdown and eventually an unwanted blood clot [55,146].

It was stated that a modification in hemostasis increases the risk of cardiovascular disease [184,185]. According to existing research, there is a correlation between decreased levels of adiponectin in the bloodstream of individuals with MASLD and an elevated risk of developing cardiovascular disease [186]. Moreover, adiponectin levels exhibited an inverse correlation with cardiovascular risk factors while demonstrating a favorable association with high-density lipoprotein-cholesterol levels [187]. Recently, the Carballo et al. (2020) study underscored the cardioprotective properties of adiponectin within the framework of ischemia–reperfusion syndrome [188]. Moreover, the research conducted by Shibata (2012) emphasizes the importance of adiponectin in offering cardiovascular protection, and this encompasses its ability to attenuate inflammatory reactions [189]. In fact, adiponectin may potentially influence hemostasis by modulating the expression and release of a number of cytokines and chemokines involved in the process of coagulation [169,182,190,191].

Coagulation proteins have been found to have a significant impact not only on the process of hemostasis but also on the development of atherogenesis [192]. The clinical presentations of atherosclerotic disease encompass coronary artery disease, peripheral arterial disease, and stroke. Atherosclerosis (AS) is a dynamic and progressive condition that results from the convergence of aberrant lipid metabolism, endothelial dysfunction, and inflammation [83,84,105,193]. An essential stage in the inflammatory process involves the penetration of monocytes into the subendothelial region of major arteries and their subsequent transformation into tissue macrophages. The activation and functioning of these macrophages are regulated by the cytokines present in the inflammatory environment of the atherosclerotic lesion [194]. MASLD exhibits a strong correlation with AS and appears to serve as an early risk factor for the development of AS [195]. In this context, it was shown that human recombinant adiponectin could stop macrophages from becoming active cells, stop macrophages from releasing TNF-α, and reduce the number of adhesion molecules that become evident on endothelial cells in a cultured cell model [196]. Consequently, it is plausible to suggest that adiponectin may exhibit anti-atherogenic characteristics.

### 3.3. Leptin

The adipokine leptin is primarily synthesized and released from adipose tissue, where it is then transported into the bloodstream [197]. Leptin is responsible for satiety, and its primary role is to serve as a negative feedback mechanism by relaying information to the hypothalamus regarding the quantity of fat stored in the periphery [197]. Individuals with obesity have a phenomenon known as central leptin resistance, resulting in elevated levels of leptin [198]. Leptin has been characterized as a regulator of various physiological mechanisms, including lipid and glucose metabolism, as well as both innate and adaptive immune responses [198,199]. Polyzos et al. conducted a meta-analysis to provide a comprehensive overview of the current understanding of the role of leptin in MASLD. After analyzing 33 studies with a total population of 2612 individuals (775 controls and 1837 MASLD patients), they found that patients diagnosed with MASLD or metabolic dysfunction-associated steatohepatitis (MASH) have higher levels of leptin in their bloodstream [200].

There exists a noteworthy correlation between leptin and indicators of active coagulation. An illustration of this can be seen in a cohort clinical study conducted in the Netherlands [201]. The study found a significant link between blood leptin levels and the concentrations of coagulation factors VIII and IX [201]. This suggests that higher levels of leptin may cause an imbalance in the coagulation system, resulting in a preference that promotes the production of blood clots. Another clinical study has shown that obese women had significantly elevated levels of coagulation activation markers, such as VWF and leptin, and these levels decreased in correlation with adipose tissue reduction following weight loss [202]. It has also been reported that increased blood leptin levels could induce the dysregulation of tissue factor and plasma activation inhibitor-1, both of which play a significant role in the development of a procoagulant state [203,204]. Bełtowski found that leptin could negatively influence blood pressure and could induce the development of arterial hypertension, both of which are strongly linked to coagulation abnormalities [205]. Previous studies have shown that leptin can trigger platelet activation and aggregation in controlled laboratory settings [206,207]. This finding implies that leptin may have the potential to induce blood clot formation, which may increase the risk of developing thrombosis. In a recent study, a positive association was seen between the severity of MASLD and MASH and the concentrations of the hormone leptin. Additionally, an elevation in platelet activation and aggregation was identified, suggesting a potential role in the promotion of arterial thrombosis [33]. Payne et al. (2014) have emphasized the role of leptin in the progression of atherosclerosis, endothelial dysfunction, and neointimal hyperplasia [208]. These processes are crucial in the development of cardiovascular diseases [105,192]. Moreover, Schäfer et al. (2014) have provided more evidence to substantiate this claim, illustrating that leptin facilitates the occurrence of arterial and venous thrombosis through multiple pathways, such as platelet activation and the modulation of prothrombotic proteins [209].

Leptin could have the potential to exert an influence on hemostasis in MASLD through many pathways. Potential mechanisms encompass the involvement of leptin in insulin resistance, inflammation, and its correlation with metabolic syndrome. Leptin and insulin have a complex relationship with each other [210]. Leptin can directly affect insulin levels in the islet cells, while insulin can stimulate the secretion of leptin by adipocytes [210]. When there is hyperinsulinemia, peripheral leptin resistance can occur, and vice versa [210]. It is widely assumed that the higher levels of leptin found in patients with MASLD contribute significantly to the development of chronic inflammation and reduced endothelial function [176,211,212]. These factors are well known to play a crucial role in endothelial dysfunction (ED) [107,114]. The presence of ED, characterized by impaired vasodilation and increased synthesis of adhesion molecules, has the capacity to promote platelet adhesion and activation, leading to the development of a procoagulant state in MASLD [113,206]. The study conducted by Ding et al. (2016) has provided evidence that individuals with chronic kidney disease exhibit increased levels of leptin, which in turn contributes to the impairment of endothelial functions [213]. There was a link between high levels of leptin hormone and ED due to leptin-mediated sympathetic activation [214,215]. Manuel-Apolinar (2013) et al. have found additional evidence to establish the link between hyperleptinemia and ED [216], demonstrating that leptin has a role in upregulating the expression of adhesion molecules and cyclooxygenase 2, hence contributing to vascular abnormalities [216]. Collectively, the aforementioned discoveries indicate that leptin is implicated in the development of ED and may hold promise as a target for therapeutic therapies. Previous studies have demonstrated an increase in leptin levels among individuals diagnosed with proliferative diabetic retinopathy (PDR) [217,218], a pathological condition characterized by the aberrant growth of blood vessels in the retina [219]. Leptin has been recognized as a plausible angiogenic agent in the context of PDR, as it was observed to induce the formation of neovascularization [220,221]. This observation serves to underscore the potential pro-coagulant effects of leptin.

As aforementioned, in MASLD, metabolic inflammation is crucial [144,145]. Hemostasis and inflammation are closely connected pathophysiologic processes [161,222,223]. Inflammation activates the hemostatic system, which greatly affects inflammatory activity [161,222,223]. Thus, the hemostatic system and inflammatory cascade create an inflammation–hemostasis loop [161,222,223]. In this context, several cytokines have been considered to be significant factors contributing to the development and progression of inflammation in MASLD, such as interleukin-1, interleukin -6, and tumor necrosis factor-α [161]. It was demonstrated that leptin could stimulate the synthesis of these cytokines, [211,224,225]. Therefore, the relationship between leptin and these cytokines provides more evidence for the involvement of leptin in the coagulation process [223,226].

The existing body of research about the correlation between leptin and hemostasis is still in its early stages, requiring further inquiries to fully understand the underlying mechanisms. The aforementioned studies underscore the intricate connection between leptin and the hemostasis system, indicating that leptin could potentially function as a procoagulant agent in specific circumstances. Hence, it is plausible that leptin may have a role in the development of hemostatic disorders in MASLD also due to its connections with obesity, a condition frequently characterized by dysregulation of leptin.

### 3.4. Resistin

The adipokine resistin is predominantly produced by adipose tissue, along with inflammatory cells such as macrophages and monocytes, as well as hepatic stellate cells [227,228]. Human resistin is a mediator of inflammation [227,228]. Resistin potentially plays a pivotal role in the etiology of MASLD [32,229,230]. MASLD patients have high serum levels of resistin [32,229,230], directly correlated with the severity of liver fibrosis [231,232]. Moreover, MASLD patients exhibited a diminished response to resistin in hepatic myeloid cells and T-lymphocytes; this decline is indicative of an inability to maintain redox homeostasis, a risk factor for the severity of MASLD [233]. MASLD is associated with metabolic syndrome [139]. It was found that there exists a significant correlation between resistin and hemostasis functions in individuals diagnosed with metabolic syndrome [234,235,236,237]. It was observed that resistin has the potential to initiate thrombotic events by modulating lipoprotein metabolism and promoting inflammation [237].

As aforementioned, endothelial cells play a crucial role in the process of blood coagulation and are responsible for maintaining overall hemostasis in the body [238]. Resistin could alter endothelial function, promoting an imbalance between coagulation and thrombosis [237,239,240]. Moreover, resistin has been demonstrated to promote angiogenesis in endothelial cells [241], and stimulate the secretion of cytokines as well as the expression of vascular adhesion molecules [242,243]. It was also reported that resistin contributes to the development of vascular lesions by inducing an increase in endothelial permeability [244]. Moreover, resistin enables monocytes to adhere to endothelial cells and promotes the production of the pro-thrombotic tissue factor [245]. Several studies have discovered that resistin also has an impact on vasoconstriction function [239,240,246]. Resistin directly could reduce endothelial-derived nitric oxide (eNOS) production and affect NO (nitric oxide) production [239,246]. Endogenous NO from eNOS is crucial for modulating platelet function in vivo [247]. eNOS appears to have a substantial impact on platelet aggregation, while iNOS and nNOS appear to play minor roles in this process [247].

An alteration in hemostasis predisposes to cardiovascular illness [184,185]. Emerging evidence suggests that cardiovascular disease is accompanied by changes in serum resistin levels [248]. Patients with acute coronary syndrome had double the serum resistin levels compared to stable angina and control patients [248]. Furthermore, blood resistin was positively correlated with indicators of inflammation and endothelial activation, such as leukocyte counts and endothelin-1 levels, in patients with unstable angina [249]. Recently, Zhou (2020) highlighted the significant impact of resistin on the development of atherosclerosis and underscored its potential as a promising therapeutic target in the context of cardiovascular disease [250].

The levels of resistin were found to be increased in individuals diagnosed with COVID-19, and this elevation was found to be correlated with the presence of cytokines and endothelial cell adhesion molecules [251]. Moreover, there was a positive correlation observed between elevated levels of resistin and unfavorable clinical outcomes in individuals diagnosed with COVID-19 [251]. Therefore, resistin could have a key role both in COVID-19 and MASLD which have, as an element in common, alterations in hemostasis.

It was reported that resistin could also play a significant role in the pathogenesis of angiogenesis-related vascular illnesses, hence potentially contributing to the development of cardiovascular disease and other angiogenic disorders [252,253]. Resistin is known to play a direct role in the process of angiogenesis [253]. It stimulates the proliferation and migration of human endothelial cells, while also facilitating the creation of capillary-like tubes [253]. The angiogenic capacity of resistin was demonstrated by its notable capability to substantially enhance the expression of vascular endothelial growth factor receptors (VEGFR-1 and VEGFR-2) and matrix metalloproteinases (MMP-1 and MMP-2) at both the mRNA and protein levels [252]. It was observed that resistin can stimulate the proliferation of human aortic smooth muscle cells (HASMC) in a manner that is dependent on the dosage administered [252]. This phenomenon has the potential to contribute to the development of vascular problems. The confirmation of the activation of both pathways by resistin was achieved by the utilization of particular inhibitors (U0126 for ERK and LY294002 for PI3K), resulting in a notable reduction in resistin-induced proliferation of human airway HASMC [252].

### 3.5. Ghrelin

Although ghrelin is not secreted by adipose tissue, it has received attention because its receptors are highly expressed in adipocytes, where ghrelin may exert a direct influence on energy metabolism [254,255]. The adipokine ghrelin is primarily synthesized and released by gastric cells [256]. Its principal functions are appetite stimulation and energy balance regulation [256,257]. Ghrelin has been shown to possess hepatoprotective properties in numerous animal models of liver damage [258,259,260,261,262]. Previous studies have demonstrated that ghrelin decreased liver damage caused by carbon tetrachloride [259,260,261], acetaminophen [262], and bile duct ligation [258], and that this hepatoprotective activity was related to its antioxidative, anti-inflammatory, and antifibrotic properties [263,264]. Moreover, it was demonstrated that ghrelin exerts an impact on insulin resistance and inflammation, both of which play crucial roles in the development of MASLD [265,266,267,268]. In this context, the *rs26802/rs696217* variants in the ghrelin gene have been observed to have a preventive effect against MASLD progression in genetic investigations [269,270]. Ghrelin exists in two distinct forms inside the bloodstream: acylated ghrelin (AG) and unacylated ghrelin (UAG) [268]. Ghelardoni et al. (2006) have reported that AG is widely distributed across many tissues inside the body [271], in addition to adipose tissue [254]. AG receptors have also been observed in high quantities in the mammalian heart, vascular smooth muscle cells, and endothelial cells (ECs), indicating the potential involvement of AG in the regulation of blood hemostasis [272] and in heart physiology [273,274,275,276]. In relation to this matter, a number of actions of AG on ECs have been identified, which encompass the inhibition of apoptosis in vascular ECs [273], the promotion of angiogenesis [277,278], and the suppression of vascular inflammation [279]. It was observed that in cultured cardiac microvascular endothelial cells, AG has the ability to enhance the processes of proliferation, migration, and nitric oxide (NO) secretion [280,281]. Furthermore, it was found that AG had a positive impact on endothelial dysfunction and increased the availability of NO in patients with metabolic syndrome [282,283]. It is noteworthy that NO formed from vascular ECs has the ability to hinder the adhesion, secretion, and aggregation of platelets [284,285]. Additionally, it was observed that NO inhibits the synthesis and release of tissue factor (TF) [286] as well as plasminogen activator inhibitor-1 (PAI-1) [287,288,289,290]. This observation supports the hypothesis that AG may potentially have an anti-thrombotic and fibrinolytic effect through its modulation of endothelial cell activity and nitric oxide generation. Furthermore, the identification of ghrelin as an endogenous ligand of the orphan receptor growth hormone secretagogue receptor 1a (GHSR-1a) has been reported to induce the release of growth hormone (GH) [271,291]. It was demonstrated that GH has the ability to improve many coagulation parameters, such as prothrombin time (PT) and activated partial thromboplastin time (aPTT) [292]. In their study, Arıcı and Cetin (2011) have demonstrated that the administration of ghrelin has a mitigating effect on coagulation dysregulation generated by carbon tetrachloride (CCl4) [261]. The administration of ghrelin prior to the application of CCl4 led to a significant decrease in PT and aPTT, accompanied by a substantial increase in fibrinogen levels, in comparison to the group that received only CCl4 treatment. Moreover, CCl4 led to a significant increase in the levels of alanine transaminase (ALT) activity, which serves as a reliable marker for liver damage, and the pre-delivery of ghrelin prior to the administration of CCl4 led to a decrease in ALT activity, similar to the levels observed in the control group [261].

Sleeve gastrectomy (SG) is a commonly used form of bariatric surgery that primarily restricts the size of the stomach [293,294]. This procedure involves the removal of a substantial amount of the stomach, including its modest curvature, resulting in the formation of a sleeve-shaped stomach [293]. Ghrelin is primarily synthesized by the cells located in the gastric fundus. Consequently, the removal of a substantial number of these ghrelin-producing cells during the treatment results in a notable reduction in the circulating levels of both AG and UAG [256,257,271]. Morsy (2020) [295] examined the impact of AG deficiency on platelet function, coagulation, and fibrinolysis in rats that underwent SG, taking into consideration the abundant expression of GHSR-1a on vascular endothelial cells and the protective effect of circulatory AG on endothelial dysfunction [273,282,283]. The subcutaneous administration of AG in rat that underwent SG resulted in a considerable inhibition of platelet aggregation and in the restoration of the normal levels of vWF and fibrinogen [295]. Interestingly, administration of AG decreased the amounts of PAI-1 and TF in the bloodstream and the aorta at the same time, while increasing the amounts of eNOS in the aorta [295]. In summary, this study concluded that AG exhibits anti-platelet, anti-coagulant, and fibrinolytic effects by acting on GHSR-1a to promote the generation of nitric oxide [295].

**Table 1:** Overview of the impact of adipokines on hemostasis processes. Adipokine levels are altered in metabolic syndrome/metabolic dysfunction-associated steatotic liver disease (MASLD), and this can interfere with the regular blood clotting process. This disruption leads to common clinical conditions such as thrombosis, atherosclerosis, and an overall higher vulnerability to cardiovascular diseases. ↑ = increased; ↓ = reduced; ⇓ = common clinical outcomes; EC = endothelial cell; TF = Tissue Factor; vWF = von Willebrand; eNOS = endothelial nitric oxide synthases.

Adipokine Levels in Metabolic Syndrome/MASLD	Pathological Outcomes	Ref.
↑ Adipose Tissue-Derived Plasminogen Activator Inhibitor-1	– ↓ fibrinolysis	[55,57,150,151]
	⇓	
	– ↑ thrombosis; ↑ atherosclerosis; ↑ cardiovascular diseases	[149,166,167,168]
↓ Adiponectin	– ↑ dysregulation of coagulation factors (*↓ PAI-1*)	[42,55,146,183]
	– ↑ dysregulated platelet activation and aggregation	[182]
	– ↑ endothelial dysfunction (*↑ EC adhesion molecules*; ↓ *eNOS*)	[172,179,180,181]
	⇓	
	– ↑ thrombosis; ↑ atherosclerosis; ↑ cardiovascular diseases	[186,187,188,189,192,196]
↑ Leptin	– ↑ coagulation activation markers (↑ factor VIII and IX, ↑ vWF)	[201,202]
	– ↑ dysregulation of coagulation factors (*↑ TF*; *↑ PAI-1*)	[203,204]
	– ↑ dysregulated platelet activation and aggregation	[206,207,209]
	– ↑ endothelial dysfunction (*↑ EC adhesion molecules*; *↑ cyclooxygenase-2*; *↑ dysregulated EC angiogenesis*)	[208,213,214,215,217,218,219,220,221]
	⇓	
	– ↑ arterial hypertension; ↑ thrombosis; ↑ atherosclerosis; ↑ cardiovascular diseases	[105,192,205,208]
↑ Resistin	– ↑ dysregulation of coagulation factors (*↑ TF*; *↑ PAI-1*)	[237,239,240,245]
	– *↑* endothelial dysfunction (*↑ EC adhesion molecules*; *↑ dysregulated EC angiogenesis*; *↑ endothelial permeability*)	[241,242,243,252,253]
	– ↑ dysregulated vasoconstriction function	[239,240,246]
	– ↑ dysregulated platelet activation and aggregation (↓ *eNOS*)	[239,246,247]
	⇓	
	– ↑ thrombosis; ↑ atherosclerosis; ↑ cardiovascular diseases	[248,249,250,251]
↓ Ghrelin	– ↑ endothelial dysfunction (*↑ EC apoptosis*; *↑ dysregulated EC angiogenesis*; *↑ vascular inflammation*; *↓ eNOS*)	[273,277,278,279,280,281,282,283]
	– ↑ platelet activation and aggregation	[282,283,284,285]
	– dysregulation of vWF (*↑*) and fibrinogen (*↑*)	[261]
	– ↑ dysregulation of coagulation factors (*↑ PAI-1*; *↑ TF*)	[280,281,282,283,287,288,289,290,295]
	⇓	
	– ↑ thrombosis; ↑ atherosclerosis; ↑ cardiovascular diseases	[273,274,275,276]

## 4. Conclusions

Metabolic dysfunction-associated steatotic liver disease (MASLD) is an intricate disorder that necessitates prompt care. MASLD is a condition that is exacerbated by disturbed hemostasis, which is a contributing factor to the progression of the disease. The role of hemostasis in the pathogenesis of MASLD is multifaceted and encompasses multiple variables. Comprehensive studies have demonstrated that adipokines have been linked to the development of metabolic syndrome and MASLD. Adipokines, mostly produced and released by adipose tissue, have a key role in controlling the sensitivity of the liver to insulin. Recently, adipokines have increasingly become recognized as regulators in the perturbed hemostasis in MASLD. Inflammation is commonly linked to both MASLD and insulin resistance (IR). The IR was found to be associated with the increase in blood glucose levels, which consequently could result in the accumulation of fatty acids and triglycerides in the liver, leading to the development of hepatic steatosis in MASLD. On the other hand, the IR in MASLD was found to correlate with alterations in the activity and concentration of adipokines. These changes could directly affect hemostasis or indirectly contribute to a vicious circle of IR, hepatic steatosis, and hyperinflammation. An increase in long-lasting inflammation may cause changes in the processes that control hemostasis at many interconnected levels. These changes could induce: (a) heightened levels of von Willebrand factor, which in turn leads to increased platelet activation and raises the risk of thrombosis; (b) elevated levels of Factor VIII and fibrinogen, which contribute to hypercoagulability, while decreased levels of antithrombin and protein C disrupt the balance between clotting and anticoagulation; (c) an increase in PAI-1 levels, coupled with a decrease in tissue activating factor antigen and tissue plasminogen activator, which results in a chronic state of hypofibrinolysis; and (d) endothelial dysfunction, which further increases the risk of thrombosis.

In summary, metabolic inflammation and liver injury are integral elements of MASLD. The interconnection of these components and the hemostasis pathway is defined by mutual impacts and the initiation of changes. Adipokines act as the shared components in this complex interaction (Figure 4).

Nevertheless, additional research is needed to fully understand the precise processes via which adipokines influence hemostasis. Hence, it is crucial to have a thorough comprehension of the function of hemostasis in MASLD and its interaction with adipokines to identify possible therapeutic targets and formulate efficient strategies for the management and treatment of MASLD. Timely implementation of therapeutic therapies can effectively impede or decelerate the advancement of MASLD, diminish comorbidities, and enhance patient outcomes.

## Data Availability

Not applicable.
